# Effects of blood flow restriction therapy in patients with knee osteoarthritis: protocol for an overview of systematic reviews

**DOI:** 10.3389/fresc.2024.1318951

**Published:** 2024-02-01

**Authors:** Felipe Alves Machado, Gustavo J. Almeida, André Luiz Maia do Vale, Alexandre Lima de Araújo Ribeiro, Graziella França Bernardelli Cipriano, Gerson Cipriano Junior, Wagner Rodrigues Martins

**Affiliations:** ^1^ Faculdade de Educacao Fisica, Universidade de Brasília, Brasília, Brazil; ^2^ Department of Physical Therapy, School of Health Professions, University of Texas Health Science Center at San Antonio, San Antonio, TX, United States; ^3^ Graduate Program in Rehabilitation Science, Faculdade de Ceilândia, Universidade de Brasília, Ceilândia, Brazil; ^4^ Department of Physiotherapy, Universidade de Brasília, Brasília, Brazil

**Keywords:** osteoarthritis, knee, blood flow restriction therapy, Kaatsu Training, resistance training, overview, systematic review

## Abstract

**Background:**

Osteoarthritis (OA) is the most common and prevalent musculoskeletal disease associated with population aging, negatively impacting function and quality of life. A consequence of knee OA is quadriceps muscle weakness. Musculoskeletal rehabilitation using low load exercises, associated with Blood Flow Restriction (BFR) may be a useful alternative to high load exercises when those cannot be tolerated. Several systematic reviews have reported inconclusive results due to discrepancies in study findings, heterogeneity of results, evaluated time points, and research questions explored.

**Objective:**

To perform an overview of systematic reviews with meta-analyses, synthesizing the most recent evidence on the effects of muscle strength training with BFR for knee OA.

**Methodology:**

Systematic reviews that include primary controlled and randomized clinical trials will be considered for inclusion. Articles will be considered only if they present a clear and reproducible methodological structure, and when they clearly demonstrate that a critical analysis of the evidence was carried out using instrumented analysis. Narrative reviews, other types of review, overviews of systematic reviews, and diagnostic, prognostic and economic evaluation studies will be excluded. Studies must include adults aged 40 years and older with a diagnosis of knee OA. Two authors will perform an electronic search with guidance from an experienced librarian. The following databases will be searched: PubMed via MEDLINE, Embase, CENTRAL (Cochrane Central Register of Controlled Trials), PEDro, Cumulative Index to Nursing and Allied Health Literature (CINAHL) via EBSCO host, Web of Science, and the gray literature. The search strategy used in the databases will follow the acronym PICOS (population, intervention, comparison, outcome, and study design). Screening (i.e., titles and abstracts) of studies identified by the search strategy will be selected using Rayyan (http://rayyan.qcri.org). The quality assessment will be performed using the “Assessment of Multiple Systematic Reviews” (AMSTAR-2) tool.

**Systematic Review Registration:**

PROSPERO, CRD42022367209.

## Background

Knee osteoarthritis (OA) is a very common and prevalent musculoskeletal chronic degenerative joint disease characterized by pain, swelling, stiffness, bone crepitus, atrophy, and muscle weakness (1), causing alterations in the cartilage metabolism and synovial inflammation, with consequent cartilage deterioration, joint space narrowing, osteophyte formation, subchondral bone sclerosis, and cystic formations (2, 3). It is estimated that OA affects approximately 250 million adults worldwide, with a prevalence correlated to the aging of the population, negatively impacting the function and quality of life of these individuals (4, 5), in addition to burdening the health system with significant costs.

These symptoms and activity limitations have been associated with non-modifiable risk factors, such as age and the female sex, in addition to modifiable factors, such as obesity, low level of physical activity, joint overload, muscle imbalance in the knee joint, and reduced weight and muscle strength in the lower limbs (6–8).

Quadriceps weakness is a characteristic finding of patients with knee OA, especially in older people, due to sarcopenia, affecting physical function through decreased strength and muscle mass (9), vascular function (10), and bone mineral density (11) that occur in aging. This muscle deficit becomes a biomechanical factor that can significantly contribute to the incidence of symptoms of knee OA and the progressive loss of joint cartilage (12–14). Several studies report that individuals with knee OA present with quadriceps muscle weakness (14–20). One study revealed that adequate quadriceps muscle strength prevents the incidence of symptom development in knee OA (13) resulting in functional improvement and decreasing the incidence and/or progression of the disease (21). Therefore, quadriceps strengthening and hypertrophy is considered as a first-line therapy (22, 23), making resistance training a common practice in OA management (23–25).

For the conditioning and muscle development of healthy people, the American College of Sports Medicine recommends a minimum of resistance training loads of 60%–70% of one repetition maximum (1RM) for strength gains, and 70%–85% of 1RM for muscle hypertrophy (26). However, training with these high loads may not be possible or may even be deleterious in individuals with knee OA.

Resistance training with low loads failed to stimulate muscle hypertrophy to the magnitude observed in resistance training with high loads after a period of 6 (27) or 8 (28) weeks, with a frequency of 3 weekly sessions. Strength adaptations were maximized with high load training (27, 28) and muscle cross-sectional area comparisons suggested that the hypertrophy and strength gains seen with low-load training are not as great as those achieved with high load training (29). However, the clinical perspective of musculoskeletal rehabilitation using low loads could be a useful strategy in situations where training using high loads are not feasible, especially in older people with knee OA (30).

Blood flow restriction (BFR) therapy has been shown to be a useful alternative to high load resistance training to improve muscle function in individuals with knee OA. BFR therapy uses inflated cuffs in the proximal region of the thigh, with occlusion pressure between 40% and 90% of the maximum, and low loads around 30% of 1 RM that can produce significant gains in muscle hypertrophy and strength (31–34). Furthermore, with regard to hypertrophy, training with BFR has shown responses comparable to those found in resistance training with high loads (35).

The physiological adaptations of muscle strength (36), and vascular (37) and pulmonary systems (38) have been reported with low-intensity aerobic exercise with BFR. Therefore, from a mechanical point of view, the hypothesis is that, in an ischemic and hypoxic environment generated by partial vascular occlusion, high levels of stress are generated along with the mechanical tension associated with exercise. Both metabolic stress and mechanical strain are described as “primary hypertrophy factors” (39) and speculated to activate other mechanisms for muscle development. These proposed mechanisms include: a systemic increase in hormone production (40, 41), healing cell stimulation (42), production of reactive oxygen species (43, 44), intramuscular anabolic/anti-catabolic signaling (45–47), and increased recruitment of fast-twitch fibers (48–50) that promote muscle tissue development.

With regard to the safety of applying BFR during exercise, in relation to hemodynamic disorders and ischemic reperfusion injury, a systematic review with meta-analysis states that with correct implementation, this technique does not present a greater risk than traditional exercise modes that do not use BFR (30, 50).

To date, a series of systematic reviews and meta-analyses (51–53) have been published investigating the evidence on the effect of muscle strength training with BFR for knee OA. These systematic reviews used various clinical outcomes, such as pain, stiffness, muscle strength and hypertrophy, functionality, mobility, and balance. However, due to discrepancies across study findings, heterogeneity of results, time points assessed, and research questions explored, these systematic reviews have reported inconclusive or contradictory results.

## Objectives of this overview

The purpose of this overview is to describe and assess the methodological quality of the current body of systematic reviews with meta-analyses. We will synthesize the best available evidence on the effects of muscle strength training with BFR in patients with knee OA, critically and systematically (see Data synthesis and reporting). This overview will examine the strengths and limitations of current evidence and discuss the applicability to clinical practice and recommendations for future research.

## Methods/design

### Protocol and registration

This is an overview of systematic reviews following the recommendations of the Cochrane Handbook (54). As per the Handbook, the unit of searching, inclusion and data extraction is the systematic review, thus we will follow their guidelines. The protocol was written using the Preferred Reporting Items for Systematic Reviews and Meta-Analyses Protocol (PRISMA-P) (55) see Supplementary Material/Appendix 1, and is registered with PROSPERO (CRD42022367209).

### Data sources and search strategy

All authors contributed to devising the search strategies for each database using a combination of subject headings and free-text keywords. Two authors (FAM and GJA) will perform the electronic search guided by a librarian with experience in database search: PubMed via MEDLINE, Excerpta Medica Database (EMBASE), CENTRAL (Cochrane Central Register of Controlled Trials), PEDro, Cumulative Index to Nursing and Allied Health Literature (CINAHL) via EBSCO host, Web of Science, Epistemonikos and gray literature via ProQuest (Brazilian Digital Library of Theses and Dissertations), and Global ETD Search (Networked Digital Library of Theses and Dissertations).

The search strategy used in all databases will follow the acronym PICOS (Participants, Interventions, Comparison, Outcome and Study Design) using standardized MeSH (Medical Subject Headings) keywords from the MeSH Database of the “National Library of Medicine” and the Boolean operators AND, OR and NOT to combine keywords for addition, alternation, or negation between terms.

To meet our objective, we will search for relevant articles combining the following terms presented in Table 1. No date or language restrictions will be applied to the initial search.

**Table 1:** Example of search strategy for pubMed via MEDLINE.

Population: #1 Osteoarthritis, Knee [MeSH Terms] #2 knee osteoarthritis [MeSH Terms] #3 osteoarthritis of knee [MeSH Terms] #4 osteoarthritis of the knee [MeSH Terms] #5 osteoarthr* [Text Word] #6=#1 OR #2 OR #3 OR #4 OR #5 Intervention: #7 blood flow restriction therapy [meSH terms] #8 Kaatsu Training [MeSH Terms] #9 vascular occlusion [Text Word] #10 “vascular occlusion training” [Text Word] #11 “blood flow restriction” [Text Word] #12 katsu [Text Word] #13=#7 OR #8 OR #9 OR #10 OR #11 OR #12 Comparison: #14 resistance training [meSH terms] #15 strength training [MeSH Terms] #16 exercise therapy [MeSH Terms] #17 exercise therapies [MeSH Terms] #18 “exercise program” [Text Word] #19 “exercise programs” [Text Word] #20=#14 OR #15 OR #16 OR #17 OR #18 OR #19 Outcomes: #21 pain [MeSH Terms] #22 chronic pain [meSH terms] #23 edema [meSH terms] #24 range of motion, articular [meSH terms] #25 range of motion [meSH terms] #26 Hypertrophy [MeSH Terms] #27 muscle strength [meSH terms] #28 muscle strength dynamometer [meSH terms] #29 muscle strength dynamometers [meSH terms] #30 isometric contraction [meSH terms] #31 isometric contractions [meSH terms] #32 physical functional performance [meSH terms] #33 functional performance [meSH terms] #34 functional performances [meSH terms] #35 physical performance [MeSH Terms] #36 physical performances [MeSH Terms] #37 physical fitness [MeSH Terms] #38 chronic pains [meSH terms] #39 joint range of motion [meSH terms] #40 joint flexibility [meSH terms] #41 passive range of motion [meSH terms] #42 quality of life [meSH terms] #43 life quality [meSH terms] #44 health-related quality of life [meSH terms] #45 health related quality of life [meSH terms] #46 HRQOL [MeSH Terms] #47 swelling [text word] #48 effusion [text word] #49 isokinetic [text word] #50 functionally-impaired [text word] #51 “functionally impaired” [text word] #52=#21 OR #22 OR #23 OR #24 OR #25 OR #26 OR #27 OR #28 OR #29 OR #30 OR #31 OR #32 OR #33 OR #34 OR #35 OR #36 OR #37 OR #38 OR #39 OR #40 OR #41 OR #42 OR #43 OR #44 OR #45 OR #46 OR #47 OR #48 OR #49 OR #50 OR #51 () Study design: #53 systematic review [meSH terms] #54 systematic reviews as Topic [MeSH Terms] #55 “systematic review” [All Fields] #56 meta analysis [MeSH Terms] #57 meta analysis as topic [MeSH Terms] #58 “meta analysis” [All Fields] #59=#53 OR #54 OR #55 OR #56 OR #57 OR #58 () Combining PICOS elements: #60=#6 AND #13 AND #20 AND #52 AND #59

### Selection of reviews

**Figure 1:**
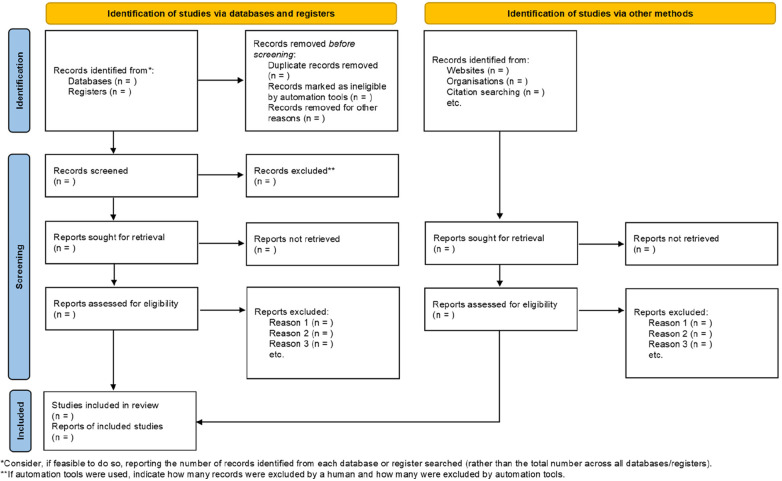
Schematic for PRISMA 2020 flow diagram of the screening process—identification of studies via databases, registers and other methods.

The results identified by the search strategy in the databases will be exported and saved into the Rayyan platform (http://rayyan.qcri.org), where two independent and blind reviewers (FAM and GJA) will perform the initial removal of duplicate and non-relevant papers based on the abstracts and titles. A full text will be acquired for those that meet the inclusion criteria. It is important to note that evidence from primary studies will not be considered. At the end of screening in Ryyan, blinding of reviewers will be opened and disagreements in study selection will be resolved by consensus among the initial reviewers and, if necessary, decided by a third reviewer (WRM). The full text documents will then be examined for eligibility. In addition, the reference lists of the selected reviews will be consulted to find possible additional systematic reviews. The anticipated study screening process is shown in Figure 1 and Supplementary Material/Appendix 2.

### Types of reviews

Systematic reviews of studies such as controlled and randomized clinical trials will be included. Articles will be deemed to be systematic reviews if they present a clear and reproducible methodological structure. Also, they must clearly demonstrate that a critical analysis of the evidence was conducted, made available through instrumented analysis [i.e., search strategy, risk of bias, and certainty of evidence and the strength of recommendations via “Grading of Recommendations Assessment, Development and Evaluation” (GRADE)]. Narrative reviews, other types of reviews, and other overviews of systematic reviews will be excluded. Diagnostic, prognosis, and economic evaluation studies will also be excluded.

### Types of participants

Studies will be selected that include patients over 40 years based on cartilage lesions in arthroscopy, magnetic resonance imaging evidence of cartilage or meniscus damage, and/or bone marrow lesions of the subchondral bone, symptoms (clinical examination—joint line tenderness and/or crepitus, knee pain without any recent trauma associated with joint stiffness), the presence of clinical risk factors (e.g., family history of OA, metabolic syndrome, malalignment and/or leg length discrepancy), patient reported outcomes [i.e., Knee Injury and Osteoarthritis Outcome score (KOOS) for defining pain and functional limitation], and Kellgren-Lawrence grade 0–1 (56–58).

In addition, the intervention needs to have been performed using strength training with vascular occlusion as a tool during the rehabilitation process. We will exclude studies in which intervention was applied to athletes and/or patients in the postoperative period.

Data extraction will include characteristics of the populations included in each eligible systematic review. This information will be discussed and interpreted accordingly.

### Types of intervention

We will include systematic reviews that used resistance training with BFR for the quadriceps muscles in open kinetic chain exercises (i.e., leg extension) and closed kinetic chain exercises (i.e., leg press, squat, or semi-squat) in individuals with knee OA. Studies with a weekly frequency of 2 or more treatment sessions for at least 4 weeks will be selected.

### Types of outcomes

#### Primary outcomes

The primary outcomes of interest are self-reported pain, knee function, muscle strength, and hypertrophy measured in the short and medium term. When applicable, these results will be summarized according to the type of BFR intervention (e.g., exercise-based rehabilitation programs with BFR using high or low intensity), assessment time point and duration of follow-up (i.e., 4, 6, or 8 weeks after intervention).

#### Secondary outcomes

Secondary outcomes will include the design of the rehabilitation programs using BFR; details on specific patient populations examined in each included review; rate of adverse events associated with the intervention; and effect of intervention on other outcome domains, such as range of motion and health-related quality of life, when reported.

### Data extraction and analysis

Data extraction and analysis will be conducted in accordance with the guidelines of the Cochrane Handbook of Systematic Reviews of Interventions. The full texts of included reviews will be retrieved. Two review authors (FAM and GJA) will independently extract descriptive and outcome data from each included review. A third review author (WRM) will arbitrate if discrepancies cannot be resolved by consensus. A bespoke data extraction form will be designed, tested, and used to record review features, including the purpose and rationale, types and numbers of studies included in the review, population(s), intervention(s), comparator(s), results (including beneficial and harmful effects, and reported adverse events), whether or not a meta-analysis was performed and the date of the last search, and methods for evaluating the quality of the studies. In case we include more than one review containing the same studies, we will examine the review question of each article, the comparisons explored, the date of the final search and key aspects of methodological quality (e.g., types of studies included and assessment of risk of bias) and will list the individual studies included in each review. This approach will enable identification of studies included in one review, and not in the other. Using this data, we will determine which of the reviews to include to contribute data to the results, based on the review with most current search strategy that included the most recent trials.

### Management of overlapping systematic reviews

It is possible that the systematic reviews included address a similar research question and the primary studies are the same, so we will take this factor into account in the data analysis. If we find overlapping systematic reviews, we will follow the Cochrane Handbook recommendations to include all non-overlapping systematic reviews and, for a group of overlapping reviews, the most recent, highest quality, most relevant or most comprehensive systematic review will be included (59). Thus, we will avoid double counting of data by ensuring that the findings of each primary study are extracted separately. A citation matrix will be constructed to visually demonstrate the amount of overlapping and the “corrected covered area” will be calculated indicating the degree of overlapping in the overview.

### Assessment of methodological quality of included reviews

Two reviewers (FAM and GJA) will independently assess the quality of the reports and the methodological quality of the included reviews using the Preferred Reporting Items for Systematic Reviews and Meta-Analyses (PRISMA) checklist (60) and the Assessment of Multiple Systematic Reviews (AMSTAR-2) tool (61), respectively. We will report the PRISMA quality ratio (number of items reported/27 checklist items *100%). The AMSTAR-2 contains 16 items that assess the methodology used in a systematic review. Each item is scored as yes, no, partially yes, and not applicable/NA. There is no total score. The presence of failures and weaknesses translates into general confidence in the results of the systematic review. Overall confidence is rated as “critically low”, “low”, “moderate”, or “high”. Based on AMSTAR-2 scores, the quality of the systematic reviews will be classified as high methodological quality (score of 8–11), as medium quality (score of 4–7), and as low quality (score of 0–3) (61).

### Data synthesis and reporting

Data will be presented as a narrative synthesis, with textual commentary supplemented with the use of summary tables and figures to enhance clarity of reporting (62). We will document primary and secondary outcomes of each intervention comparison from the included reviews, as well as the number of studies and number of participants included in each comparison. Data (when reported in the review) will be described as mean difference (or standardized mean difference), 95% confidence intervals, and *I^2^* statistic for heterogeneity (63). We will synthesize key information pertaining to the quality of evidence, and documented eligibility criteria, study characteristics, and the primary outcome of each review. Flow diagrams will be used to summarize the study selection process. Finally, reasons for excluding reviews will be described.

### Sub-group analysis

Depending on the amount of information provided by the reviews and number of participants included, we plan to analyze key functional outcomes according to patient characteristics (e.g., age and sex) and intensity of intervention (i.e., BFR associated with high vs. low intensity resistance training).

## Discussion

### Expected significance of the study

The findings of this overview of systematic reviews on the effects of BFR in patients with knee OA will potentially have implications for clinical practice, research, and the future development and/or updating of guidelines. Our results are intended to provide greater clarity and synthesis of the available evidence on this intervention technique and its effectiveness to improve symptom-related and functional outcomes in this population. Such information is likely to impact existing and planned resource allocation in the clinical setting, inform the direction of future research, including randomized controlled trials on the effectiveness of BFR training, and support guideline recommendations. The conclusions in this overview will highlight outcomes that demonstrate clear benefits and those for which there is no clear evidence. If sufficient data are available, our findings may also add clarity to the “dose” of the BFR intervention (i.e., type, intensity of exercise load, intensity of vascular occlusion pressure, and duration), as well as circumstances under which any adverse events or harm were reported as a consequence of the intervention.

### Potential limitations of overview design

We plan to strictly follow the approach outlined by the Cochrane Collaboration to undertake an overview of systematic reviews (64). We will note when included systematic reviews are out of date and identify any relevant new studies that have been published after the date of the last reported systematic review search. However, we will not formally consider recent articles not included in previous systematic reviews. We are not planning to undertake a new systematic review within our general framework (64). Our discussion will focus on the current state of evidence related to BFR in patients with knee OA based on systematic reviews.

## Conclusion

The proposed overview will summarize the current knowledge about the role of BFR therapy in patients with knee OA along with the strength of evidence from outcomes reported in the included systematic reviews. This overview will provide the clinicians and researchers with some level of certainty on the effects of BFR therapy on several outcomes in individuals with knee OA. Furthermore, the overview may shed light on future directions for systematic reviews and possibly new research studies.

## Data Availability

The raw data supporting the conclusions of this article will be made available by the authors, without undue reservation.
