# Supplementary material for: Effects of blood flow restriction therapy in patients with knee osteoarthritis: protocol for an overview of systematic reviews
Source: Front Rehabil Sci. 2024 Feb 1;5:1318951. doi: 10.3389/fresc.2024.1318951 (PMC10867121; doi:10.3389/fresc.2024.1318951)
Supplement: Supplementary file 2 [file Table2.docx]

**Supplementary Material 2.**

PICOS Strategy and Search Strategy of all databases

PICOS elements to initially guide the search strategy.

**Population**: Osteoarthritis of knee (Osteoarthritis, Knee [MeSH])

**Intervention**: strength training with vascular occlusion (Blood Flow Restriction Therapy [MeSH]; Kaatsu Training [MeSH])

**Comparison**: Resistance Training [MeSH]

**Outcome**: Pain [MeSH], swelling, range of motion (Range of Motion, Articular [MeSH]), quadriceps strength (Muscle Strength [MeSH]), Hypertrophy [MeSH], Quality of Life [MeSH]

**Study Design**: Systematic Review [MeSH], Meta-Analysis [MeSH]

Search Strategy

**Database 1: Pubmed (Medline)**

- Population:

#1 Osteoarthritis, Knee [MeSH Terms]

#2 knee osteoarthritis [MeSH Terms]

#3 osteoarthritis of knee [MeSH Terms]

#4 osteoarthritis of the knee [MeSH Terms]

#5 osteoarthr* [Text Word]

#6 = #1 OR #2 OR #3 OR #4 OR #5

- Intervention:

# #7 Blood Flow Restriction Therapy [MeSH Terms]

# # 8 Kaatsu Training [MeSH Terms]

# 9 vascular occlusion [Text Word]

#10 “vascular occlusion training” [Text Word]

#11 “blood flow restriction” [Text Word]

#12 katsu [Text Word]

#13 = #7 OR #8 OR #9 OR #10 OR #11 OR #12

- Comparison:

# #14 Resistance Training [MeSH Terms]

#15 strength training [MeSH Terms]

#16 exercise therapy [MeSH Terms]

#17 exercise therapies [MeSH Terms]

#18 “exercise program” [Text Word]

#19 “exercise programs” [Text Word]

#20 = #14 OR #15 OR #16 OR #17 OR #18 OR #19

- Outcomes:

#21 pain [MeSH Terms]

# #22 chronic pain [MeSH Terms]

# #23 edema [MeSH Terms]

# #24 Range of Motion, Articular [MeSH Terms]

# #25 range of motion [MeSH Terms]

#26 Hypertrophy [MeSH Terms]

# #27 muscle strength [MeSH Terms]

# #28 muscle strength dynamometer [MeSH Terms]

# #29 muscle strength dynamometers [MeSH Terms]

# #30 isometric contraction [MeSH Terms]

# #31 isometric contractions [MeSH Terms]

# #32 physical functional performance [MeSH Terms]

# #33 functional performance [MeSH Terms]

# #34 functional performances [MeSH Terms]

#35 physical performance [MeSH Terms]

#36 physical performances [MeSH Terms]

#37 physical fitness [MeSH Terms]

# #38 chronic pains [MeSH Terms]

# #39 joint range of motion [MeSH Terms]

# #40 joint flexibility [MeSH Terms]

# #41 passive range of motion [MeSH Terms]

# #42 quality of life [MeSH Terms]

# #43 life quality [MeSH Terms]

# #44 health-related quality of life [MeSH Terms]

# #45 health related quality of life [MeSH Terms]

#46 HRQOL [MeSH Terms]

# #47 swelling [Text Word]

# #48 effusion [Text Word]

# #49 isokinetic [Text Word]

# #50 functionally-impaired [Text Word]

# #51 “functionally impaired” [Text Word]

# #52 = #21 OR #22 OR #23 OR #24 OR #25 OR #26 OR #27 OR #28 OR #29 OR #30 OR #31 OR #32 OR #33 OR #34 OR #35 OR #36 OR #37 OR #38 OR #39 OR #40 OR #41 OR #42 OR #43 OR #44 OR #45 OR #46 OR #47 OR #48 OR #49 OR #50 OR #51

- Study Design:

# #53 systematic review [MeSH Terms]

#54 systematic reviews as Topic [MeSH Terms]

#55 “systematic review” [All Fields]

#56 meta analysis [MeSH Terms]

#57 meta analysis as topic [MeSH Terms]

#58 “meta analysis” [All Fields]

#59 = #53 OR #54 OR #55 OR #56 OR #57 OR #58

Combining PICOS elements:

#60 = #6 AND #13 AND #20 AND #52 AND #59

**Database 2: EMBASE**

- Population:

#1: 'knee osteoarthritis'/exp

#2 osteoarthr*

#3 = #1 OR #2

- Intervention:

# #4 'blood flow restriction training'/exp

# # 5 ‘kaatsu training’

# 6 'vascular occlusion'

#7 ‘vascular occlusion training’

#8 'blood flow restriction'

#9 'kaatsu'

#10 = #4 OR #5 OR #6 OR #7 OR #8 OR #9

- Comparison:

# #11 'resistance training'/exp

#12 'muscle strength'/exp

#13 'kinesiotherapy'/exp

#14 ‘exercise therapies’

#15 'exercise program'

#16 'exercise programs'

#17 = #11 OR #12 OR #13 OR #14 OR #15 OR #16

- Outcomes:

#18 'pain'/exp

# #19 'chronic pain'/exp

# #20 'edema'/exp

# #21 'range of motion'/exp

# #22 'hypertrophy'/exp

#23 'muscle strength'/exp

# #24 'dynamometer'/exp

# #25 'muscle isometric contraction'/exp

# #26 'physical performance'/exp

# #27 'fitness'/exp

# #28 'quality of life'/exp

# #29 'joint swelling'/exp

# #30 'effusion'/exp

# #31 'isokinetic exercise'/exp

# #32 = #18 OR #19 OR #20 OR #21 OR #22 OR #23 OR #24 OR #25 OR #26 OR #27 OR #28 OR #29 OR #30 OR #31

- Study Design:

# #33 'systematic review'/exp

#34 'systematic review (topic)'/exp

#35 'meta analysis'/exp

#36 'meta analysis (topic)'/exp

#37 = #33 OR #34 OR #35 OR #36

Combining PICOS elements:

#38 = #3 AND #10 AND #17 AND #32 AND #37

**Database 3: CENTRAL (Cochrane Library)**

- Population:

#1 MeSH: “Osteoarthritis, Knee”

#2 MeSH: “knee osteoarthritis”

#3 MeSH: “osteoarthritis of knee”

#4 MeSH: “osteoarthritis of the knee”

#5 Text word: osteoarthr*

#6 = #1 OR #2 OR #3 OR #4 OR #5

- Intervention:

# #7 MeSH: “Blood Flow Restriction Therapy”

# # 8 MeSH: “Kaatsu Training”

# 9 Text word: “vascular occlusion”

#10 Text word: “vascular occlusion training”

#11 Text word: “blood flow restriction”

#12 Text word: kaatsu

#13 = #7 OR #8 OR #9 OR #10 OR #11 OR #12

- Comparison:

# #14 MeSH: “Resistance Training”

#15 MeSH: “strength training”

#16 MeSH: “exercise therapy”

#17 MeSH: “exercise therapies”

#18 Text word: “exercise program”

#19 Text word: “exercise programs”

#20 = #14 OR #15 OR #16 OR #17 OR #18 OR #19

- Outcomes:

#21 MeSH: pain

# #22 MeSH: “chronic pain”

# #23 MeSH: edema

# #24 MeSH: “Range of Motion, Articular”

# #25 MeSH: “range of motion”

#26 MeSH: “Hypertrophy”

# #27 MeSH: “muscle strength”

# #28 MeSH: “muscle strength dynamometer”

# #29 MeSH: “muscle strength dynamometers”

# #30 MeSH: “isometric contraction”

# #31 MeSH: “isometric contractions”

# #32 MeSH: “physical functional performance”

# #33 MeSH: “functional performance”

# #34 MeSH: “functional performances”

#35 MeSH: “physical performance”

#36 MeSH: “physical performances”

#37 MesH: “physical fitness”

# #38 MeSH: “chronic pains”

# #39 MeSH: “joint range of motion”

# #40 MeSH: “joint flexibility”

# #41 MeSH: “passive range of motion”

# #42 MeSH: “quality of life”

# #43 MeSH: “life quality”

# #44 MeSH: “health-related quality of life”

# #45 MeSH: “health related quality of life”

#46 MeSH: HRQOL

# #47 Text word: swelling

# #48 Text word: effusion

# #49 Text word: isokinetic

# #50 Text word: “functionally-impaired”

# #51 Text word: “functionally impaired”

# #52 = #21 OR #22 OR #23 OR #24 OR #25 OR #26 OR #27 OR #28 OR #29 OR #30 OR #31 OR #32 OR #33 OR #34 OR #35 OR #36 OR #37 OR #38 OR #39 OR #40 OR #41 OR #42 OR #43 OR #44 OR #45 OR #46 OR #47 OR #48 OR #49 OR #50 OR #51

Combining PICOS elements:

#53 = #6 AND #13 AND #20 AND #52

**Database 4: Web of Science**

- Population:

#1 TS=(“Osteoarthritis, Knee”)

#2 TS=(“knee osteoarthritis”)

#3 TS=(“osteoarthritis of knee”)

#4 TS=(“osteoarthritis of the knee”)

#5 TS=(osteoarthr*)

#6 = #1 OR #2 OR #3 OR #4 OR #5

- Intervention:

# #7 TS=(“Blood Flow Restriction Therapy”)

# # 8 TS=(“Kaatsu Training”)

# 9 TS=(“vascular occlusion”)

#10 TS=(“vascular occlusion training”)

#11 TS=(“blood flow restriction”)

#12 TS=(katsu)

#13 = #7 OR #8 OR #9 OR #10 OR #11 OR #12

- Comparison:

# #14 TS=(“Resistance Training”)

#15 TS=(“strength training”)

#16 TS=(“exercise therapy”)

#17 TS=(“exercise therapies”)

#18 TS=(“exercise program”)

#19 TS=(“exercise programs”)

#20 = #14 OR #15 OR #16 OR #17 OR #18 OR #19

- Outcomes:

#21 TS=(pain)

# #22 TS=(“chronic pain”)

# #23 TS=(edema)

# #24 TS=(“Range of Motion, Articular”)

# #25 TS=(“range of motion”)

#26 TS=(“Hypertrophy”)

# #27 TS=(“muscle strength”)

# #28 TS=(“muscle strength dynamometer”)

# #29 TS=(“muscle strength dynamometers”)

# #30 TS=(“isometric contraction”)

# #31 TS=(“isometric contractions”)

# #32 TS=(“physical functional performance”)

# #33 TS=(“functional performance”)

# #34 TS=(“functional performances”)

#35 TS=(“physical performance”)

#36 TS=(“physical performances”)

#37 TS=(“physical fitness”)

# #38 TS=(“chronic pains”)

# #39 TS=(“joint range of motion”)

# #40 TS=(“joint flexibility”)

# #41 TS=(“passive range of motion”)

# #42 TS=(“quality of life”)

# #43 TS=(“life quality”)

# #44 TS=(“health-related quality of life”)

# #45 TS=(“health related quality of life”)

#46 TS=(HRQOL)

# #47 TS=(swelling)

# #48 TS=(effusion)

# #49 TS=(isokinetic)

# #50 TS=(“functionally-impaired”)

# #51 TS=(“functionally impaired”)

# #52 = #21 OR #22 OR #23 OR #24 OR #25 OR #26 OR #27 OR #28 OR #29 OR #30 OR #31 OR #32 OR #33 OR #34 OR #35 OR #36 OR #37 OR #38 OR #39 OR #40 OR #41 OR #42 OR #43 OR #44 OR #45 OR #46 OR #47 OR #48 OR #49 OR #50 OR #51

- Study Design:

# #53 TS=(“systematic review”)

#54 TS=(“Systematic Reviews as Topic”)

#55 TS=(“meta-analysis”)

#56 TS=(“Meta Analysis as Topic”)

#57 = #53 OR #54 OR #55 OR #56

Combining PICOS elements:

#58 = #6 AND #13 AND #20 AND #52 AND #57

**Database 5: CINAHL e Sports Discuss (Ebsco Host)**

- Population:

#1 MeSH: “Osteoarthritis Knee”

#2 MeSH: “knee osteoarthritis”

#3 MeSH: “osteoarthritis of knee”

#4 MeSH: “osteoarthritis of the knee”

#5 Text word: osteoarthr*

#6 = #1 OR #2 OR #3 OR #4 OR #5

- Intervention:

# #7 MeSH: “Blood Flow Restriction Therapy”

# # 8 MeSH: “Kaatsu Training”

# 9 Text word: “vascular occlusion”

#10 Text word: “vascular occlusion training”

#11 Text word: “blood flow restriction”

#12 Text word: kaatsu

#13 = #7 OR #8 OR #9 OR #10 OR #11 OR #12

- Comparison:

# #14 MeSH: “Resistance Training”

#15 MeSH: “strength training”

#16 MeSH: “exercise therapy”

#17 MeSH: “exercise therapies”

#18 Text word: “exercise program”

#19 Text word: “exercise programs”

#20 = #14 OR #15 OR #16 OR #17 OR #18 OR #19

- Outcomes:

#21 MeSH: pain

# #22 MeSH: edema

# #23 MeSH: “range of motion”

#24 MeSH: “Hypertrophy”

# #25 MeSH: “muscle strength”

# #26 MeSH: “physical functional performance”

# #27 MeSH: “quality of life”

# #28 Text word: swelling

# #29 Text word: effusion

# #30 Text word: disability

# #31 Text word: Functionality

# #32 Text word: Funcion

# #33 = #21 OR #22 OR #23 OR #24 OR #25 OR #26 OR #27 OR #28 OR #29 OR #30 OR #31 OR #32

- Study Design:

# #34 MeSH: “systematic review”

#35 MeSH: “Systematic Reviews as Topic”

#36 MeSH: “meta-analysis”

#37 MeSH: “Meta Analysis as Topic”

#38 = #53 OR #54 OR #55 OR #56

Combining PICOS elements:

#38 = #6 AND #13 AND #20 AND #33 AND #38

**Database 6: PEDro (**Physiotherapy Evidence Database)

"blood flow restriction"

"kaatsu" "systematic review"

"vascular occlusion" "systematic review"

**Proquest e BDTD**

(“Blood Flow Restriction Therapy” OR “Kaatsu Training” OR “vascular occlusion” OR “vascular occlusion training” OR “blood flow restriction” OR katsu)

**Epistemonikos** (systematic review repositories)

(title:(osteoarthritis) OR abstract:(osteoarthritis)) OR (title:(osteoart*) OR abstract:(osteoart*)) OR (title:(knee) OR abstract:(knee)) AND (title:(blood flow restriction) OR abstract:(blood flow restriction)) OR (title:(kaatsu) OR abstract:(kaatsu)) OR (title:(vascular occlusion) OR abstract:(vascular occlusion)) AND (title:(systematic review) OR abstract:(systematic review)) OR (title:(meta analysis) OR abstract:(meta analysis))

**NDLTD (Global ETD Search)**

**Networked Digital Library of Theses and Dissertations**

Spelling suggestions: [*"knee osteoarthritis AND blood flow restrictions"*](http://search.ndltd.org/search.php?q=knee+osteoarthritis+AND+blood+flow+restrictions)

Spelling suggestions: [*"(osteoarthri*) AND (“blood low restriction 20therapy” OR “kaatsu baraining” OR “vascular occlusion” OR “vascular occlusion training” OR “blood flow restrictions” OR matsu) AND ("systematic review" OR metaanalysis)"*](http://search.ndltd.org/search.php?q=%28osteoarthri%2A%29+AND+%28%E2%80%9Cblood+low+restriction+20therapy%E2%80%9D+OR+%E2%80%9Ckaatsu+baraining%E2%80%9D+OR+%E2%80%9Cvascular+occlusion%E2%80%9D+OR+%E2%80%9Cvascular+occlusion+training%E2%80%9D+OR+%E2%80%9Cblood+flow+restrictions%E2%80%9D+OR+matsu%29+AND+%28%22systematic+review%22+OR+metaanalysis%29)

[*"(osteoarthri*) AND (“blood low restriction 20therapy” OR “kaatsu baraining” OR “vascular occlusion” OR “vascular occlusion training” OR “blood flow restrictions” OR matsu) AND ("systematic review" OR meta­analysis)"*](http://search.ndltd.org/search.php?q=%28osteoarthri%2A%29+AND+%28%E2%80%9Cblood+low+restriction+20therapy%E2%80%9D+OR+%E2%80%9Ckaatsu+baraining%E2%80%9D+OR+%E2%80%9Cvascular+occlusion%E2%80%9D+OR+%E2%80%9Cvascular+occlusion+training%E2%80%9D+OR+%E2%80%9Cblood+flow+restrictions%E2%80%9D+OR+matsu%29+AND+%28%22systematic+review%22+OR+meta%C2%ADanalysis%29)
